# Magnetic resonance imaging for jawbone assessment: a systematic review

**DOI:** 10.1186/s13005-024-00424-2

**Published:** 2024-04-19

**Authors:** Hian Parize, Sofya Sadilina, Ricardo Armini Caldas, João Victor Cunha Cordeiro, Johannes Kleinheinz, Dalva Cruz Laganá, Newton Sesma, Lauren Bohner

**Affiliations:** 1 Department of Cranio-Maxillofacial Surgery, University Hospital Munster, Munster, Germany; 2 Department of Prosthodontics, University of São Paulo, São Paulo, Brazil; 3 Clinic of Reconstructive Dentistry, Center of Dental Medicine, University of Zurich, Zurich, Switzerland; 4 Department of Dentistry, Federal University of Santa Catarina, R. Delfino Conti, 1240 - Trindade, Florianopolis, Florianópolis, 88040-535 SC Brazil

**Keywords:** Surgery oral, Magnetic resonance imaging, Bone Assessment, Systematic review

## Abstract

**Purpose:**

To evaluate the accuracy of magnetic resonance imaging (MRI) for jawbone assessment compared to reference-standard measurements in the literature.

**Materials and methods:**

An electronic database search was conducted in PubMed, EMBASE, Scopus, Web of Science, and the Cochrane Library in June 2022, and updated in August 2023. Studies evaluating the accuracy of MRI for jawbone assessment compared with reference-standard measurements (histology, physical measurements, or computed tomography) were included. The outcome measures included bone histomorphometry and linear measurements. The risk of bias was assessed by the Quality Assessment Tool for Diagnostic Accuracy Studies (QUADAS-2). The review was registered in the PROSPERO database (CRD42022342697).

**Results:**

From 63 studies selected for full-text analysis, nine manuscripts were considered eligible for this review. The studies included assessments of 54 participants, 35 cadavers, and one phantom. A linear measurement error ranging from 0.03 to 3.11 mm was shown. The accuracy of bone histomorphometry varies among studies. Limitations of the evidence included heterogeneity of MRI protocols and the methodology of the included studies.

**Conclusion:**

Few studies have suggested the feasibility of MRI for jawbone assessment, as MRI provides comparable results to those of standard reference tests. However, further advancements and optimizations are needed to increase the applicability, validate the efficacy, and establish clinical utility of these methods.

**Supplementary Information:**

The online version contains supplementary material available at 10.1186/s13005-024-00424-2.

## Introduction

Imaging exams are an essential complement for diagnosing and treating diseases and conditions affecting the maxillofacial region. Despite the exposure to ionizing radiation and limitations in displaying and differentiating soft tissues, intraoral radiography, extraoral radiography and cone beam computed tomography (CBCT) are commonly used to evaluate maxillofacial structures [1].

CBCT is still considered the standard imaging technique for assessing maxillofacial bones, but magnetic resonance imaging (MRI) has emerged as a potential alternative due to its superior soft tissue contrast and lack of ionizing radiation. MRI is a noninvasive diagnostic tool that generates images based on the interaction between magnetic fields and radio waves with hydrogen atoms present in the human body [2].

Advancements in MRI, primarily known for its application in soft tissue evaluation, have extended to include quantitative bone imaging and microstructural analysis [3]. For instance, the ability of MRI to detect interactions between water, fat, and blood within marrow tissues has enabled the assessment of different trabecular and cortical characteristics [4, 5]. Additionally, indirect assessment of bone mineral density (BMD) by quantifying bone mineral fat (BMF) can offer an alternative approach to the conventional X-ray imaging techniques commonly used for osteoporosis diagnosis, fracture risk prediction, and treatment planning [6]. Moreover, the application of standard pulse sequences combined with commercially available coils and MRI scanners can allow for detailed bone microarchitecture assessment [7].

A recent pilot study evaluated different trabecular bone parameters via MRI in comparison to those via microcomputed tomography (µCT). While MRI has been shown to slightly overestimate bone parameters, indicating increased density, it also exhibited statistically significant fixed linear deviations [8]. Other studies reported similar bone results using MRI and other imaging techniques, including CT [9] and CBCT [10–12].

Therefore, MRI has been suggested as a potential alternative for assessing bone quality in various dental scenarios, including dental and periodontal anatomical analysis [13], cephalometry [14] and panoramic imaging [15], preoperative diagnosis in third molar surgery [16], caries detection [17], and dental implant planning [18–20]. However, despite recent improvements, the literature on the use of MRI for jawbone assessment is scarce, and different methods for obtaining MR images are available. Therefore, the aim of this study was to evaluate the accuracy of MRI for jawbone assessment compared to reference-standard measurements in the literature.

## Materials and methods

### Protocol registration

This systematic review was performed according to the Preferred Reporting Items of Systematic Reviews and Meta-analysis (PRISMA) [21] and registered in the International Prospective Register of Systematic Reviews (PROSPERO) [22] according to the CRD42022342697 protocol [23]. The proposed focused question was “What is the accuracy of magnetic resonance imaging for jawbone assessment compared to reference-standard measurements?”.

### Eligibility criteria

The diagnostic studies (e.g., experimental, observational, clinical, animal, in vitro and ex vivo) with no language or time restriction were included following the eligibility criteria established according to the PIRD method (population, index test, reference test and diagnostic of interest) and are described as follows: population - healthy maxilla and mandible sites; index test - magnetic resonance imaging; reference test - reference-standard measurements (e.g., histology, physical measurements, or computed tomography); and diagnosis of interest - quantitative and/or bone histomorphometry measurements. The exclusion criteria for studies were as follows: evaluating maxillary and mandibular sites under deformities; traumatic, pathological, or healing conditions (these conditions can alter tissue fluid levels, potentially distorting bone assessments in MRI); not performing quantitative and/or qualitative bone histomorphometry measurements by means of MRI and reference standards; reviews, letters, abstracts, posters, research protocols, personal opinions, case reports, or technique articles; and studies in which the full-text was unavailable.

### Information sources

Five electronic databases (Medline via PubMed, EMBASE, Scopus, Web of Science, and CENTRAL [the Cochrane Central Register of Controlled Trials Cochrane Library]) were used to identify studies by two reviewers. The searches were conducted up to June 22nd, 2022, and updated on August 18th, 2023. No time or language restrictions were applied. Additionally, the reference lists of the studies included in the full-text analyses and of relevant review articles on the topic [6, 12, 16, 24–27] were manually searched.

### Search strategy

The primary search strategy was obtained and applied to the PubMed database. Later, the primary search was adapted for other databases to meet their requirements (additional file 1).

### Selection process

Duplicates were removed, and two reviewers (H.P. and S.S.) independently examined the studies by title and abstract for full-text reading. Afterwards, the eligibility criteria were applied to the studies remaining, resulting in the inclusion of studies in this review. Conflicts were settled by consensus or, if they persisted, by the judgment of a third independent review author (L. B.). All articles that did not meet the eligibility criteria were excluded and are presented in additional file 2.

### Data collection process and items

The data from the included studies were extracted in duplicate by two reviewers and cross-checked. Quantitative bone histomorphometry was regarded as the primary outcome. The definitions from the American Society for Bone and Mineral Research were adopted for bone tissue (cortical or cancellous), and the histomorphometry outcomes included linear measurements (distance between points) and structural indices (e.g., trabecular bone density, trabecular separation, and trabecular width) [28]. When needed, the authors of the included studies were contacted to obtain additional information.

### Study risk of bias assessment

The risk of bias was assessed using the Quality Assessment Tool for Diagnostic Accuracy Studies-2 (QUADAS-2) by two independent reviewers. The tool is divided into four categories: patient selection, index test, reference standard, and flow and timing. The questions were answered as “Yes”, “Unclear” or “No” according to the potential risk of bias [29].

### Certainty assessment

The certainty of evidence was assessed according to the Grading of Recommendation Assessment, Development, and Evaluation (GRADE) criteria [30] by two independent reviewers.

## Results

### Study selection

**Fig. 1:**
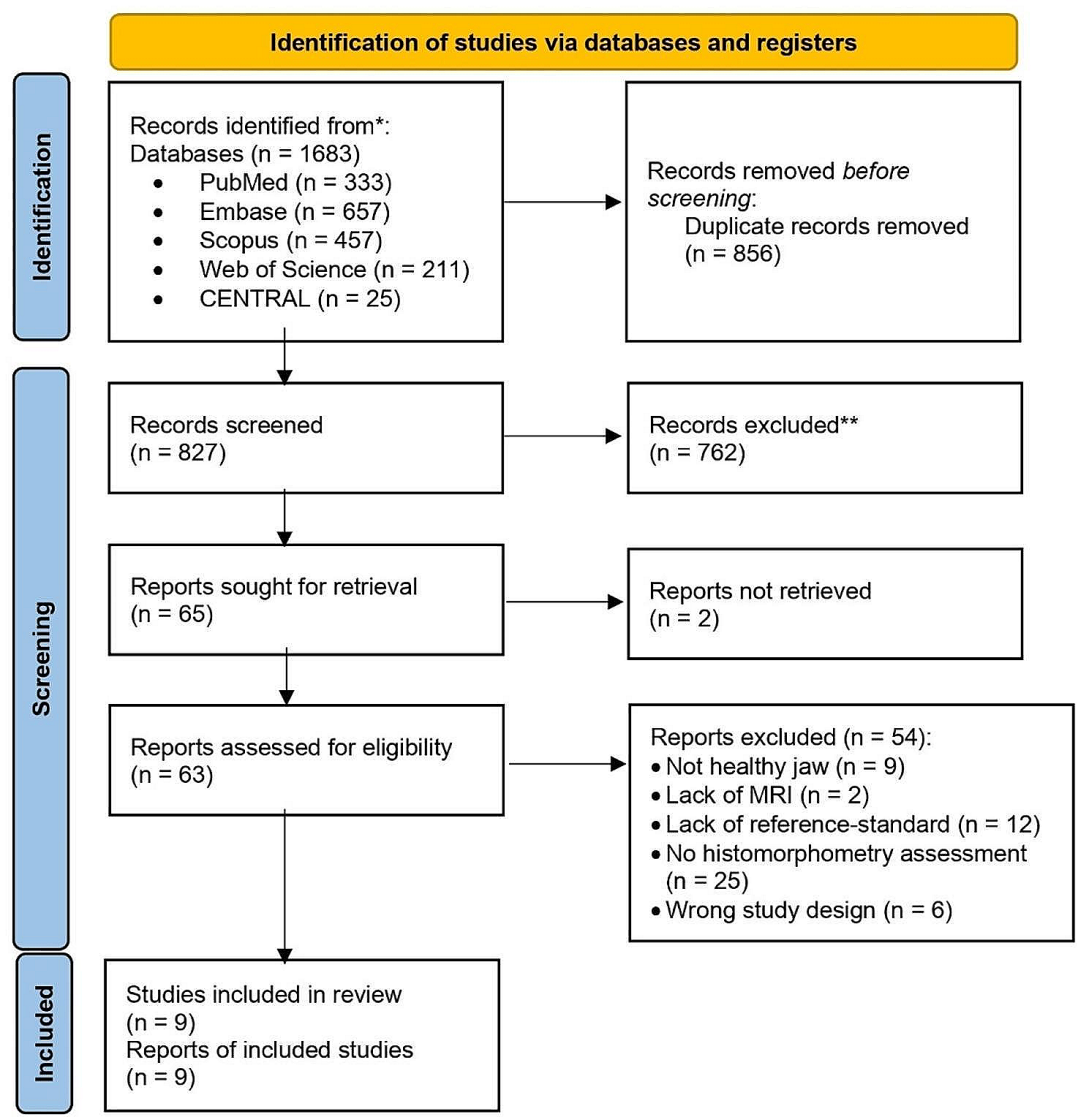
Flow chart of the screening process (PRISMA 2020)^21^

The electronic search identified 1681 articles, which were reduced to 825 after duplicate removal. Sixty-three articles were selected for full-text analysis after the title and abstract were screened. The full texts of two studies were not found; thus, they were excluded. Finally, 54 articles were excluded during this stage (reasons for exclusion are reported in additional file 2), leaving nine articles suitable for this review (Fig. 1). Cohen’s kappa coefficient for interrater reliability was 0.868.

### Study characteristics

The study characteristics are described in Table 1. In total, four in vivo [9, 31–33], three in vitro [11, 12, 34], and two ex vivo/in vitro [10, 35] studies were assessed, including a total of 54 patients, 35 cadavers and one phantom. Most of those studies evaluated the mandible [9–12, 32–35], and three studies evaluated the maxilla [10, 12, 33], mostly at posterior sites [9–12, 35]. In terms of diagnostic purposes, most studies have evaluated MRI for dental implant planning [9, 12, 31–34], two studies have focused on maxillofacial surgery planning [11, 35], and one has applied MRI for routine maxillofacial diagnosis [10]. For outcomes, most studies have evaluated bone geometric accuracy through linear measurements [9–12, 33–35] and angular measurements. Two studies assessed the trabecular structure through bone density/volume [31, 32]. The most common reference test was CT [9, 11, 34, 35], followed by digital calipers [11, 34, 35], CBCT [10, 12, 31, 33], µ-CT [31, 32] and histology [10]. Additionally, the studies differed according to the MRI device, magnetic field strength, sequence parameters, and radiofrequency coil employed.

**Table 1:** Characteristics of the included studies

First author, year	Type of participants (N)	Evaluated site	Diagnostic purpose	Magnetic field strength (MRI device)	Sequence type (MRI parameters)	Coil type and position	Reference test	No. of examiners (examiner experience)	Outcome evaluated
Aguiar, 2009^34^	Dry cadaver (5)	Anterior mandible	Geometric accuracy of cortical bone for dental implant planning	1.0 T (Signa Contour, GE HealthCare)	T2- weighted (ST 2 mm; table feed 1 mm; TR/TE 112/3.5 ms; FOV 26 cm; matrix 256 × 256 pixels; scanning time 3 min.)	Head coil	CT and digital caliper	4 (specialists in Oral and Maxillo-Facial Radiology)	Bone height
Al-Haj Husain, 2023^33^	Patients (10)	Anterior mandible and maxilla	Image quality and diagnostic accuracy of buccal bone thickness assessment for dental implant planning	3 T (Skyra, Siemens)	3D double-echo steady state (DESS) (isotropic resolution of 0.75 × 0.75 × 0.75 mm, receive bandwidth of 355 Hz/Px, FOV 242 × 242 × 78 mm^3^; acquisition matrix 320 × 320 × 104; slice oversampling 100%; no parallel acquisition; one signal average; acquisition time 12:24 min:s; TR/TE1/TE2 11.2/4.2/7.7 ms; flip angle 30°; selective water excitation.)	Siemens standard 64 channel head-and-neck coil.	CBCT	2 (oral surgeon and oral and maxillofacial radiology)	Image quality, image artifact, and cortical bone thickness
Choi, 2022^31^	Bone specimens (21) from patients (18)	NR	Structural assessment (indirect assessment of trabecular bone density/volume) for dental implant planning	14 T (Magnex interfaced to a Bruker BioSpin (Billerica, MA) Avance III HD console)	3D gradient-echo (TR/TE 48.6/2.6ms; matrix 256 × 256 × 256 pixels; scanning time ~ 14 h.)	Custom build Bruker coil inner/outer diameter 10/40 mm/NR	CBCT and micro-CT	NR	MRI: bone mineral adipose tissue volume; micro-CT: BV/TV; CBCT: radiodensity.
Cortes, 2018^32^	Bone specimens (7) from patients (7)	Mandible	Structural assessment (indirect assessment of trabecular bone density/volume) for dental implant planning	15 T (130-mm horizontal bore magnet (Agilent, Yarnton), 60-mm ID gradient insert (Resonance Research Inc.) with 2370 mT/m maximum gradient, interfaced to a Siemens console (Siemens))	3D gradient-echo pulse (TE/TR 3.3/50 ms; receiver bandwidth 255-Hz; flip angle 25°; 16 averages; FOV 7.5 × 7.5 × 7.5 mm; matrix 128 × 128 × 128 pixels; voxel 59 m³; scanning time 32:07 min.)	Custom-built 13-mm inner diameter loop-gap probe/NR	micro-CT	2/(PhD in oral radiology and postdoctoral fellow trained in radiology and MRI; PhD student and master in biomaterials)	MRI: bone marrow fat volume and BV/TV; micro-CT: BV/TV
Deng, 2014^11^	Fresh cadaver (1)	Posterior mandible	Geometric accuracy of inferior alveolar nerve and cortical bone for maxillofacial surgery planning	3 T (Magnetom Trio, Siemens)	Fast gradient-echo (TR/TE 2,3/3.67 ms; flip angle 10°; matrix 448 × 448 pixels; FOV 226 × 226 mm.)	Supine conventional position	CT and digital caliper	NR	Mandibular nerve canal to cortical bone linear measurement
Flügge, 2016^10^	Patients (2) and dry cadaver (1)	Posterior maxilla and mandible	Imaging and geometric accuracy of hard and soft tissues for maxillofacial diagnosing	3 T (TIM Trio, Siemens)	Gradient-echo fast low flip angle shots (FLASH) (In vivo, mandible: Matrix 64 × 64 × 28 mm (115 cm³); voxel 250 × 250 × 500 μm³; TE/TR 4.2/11 ms; flip angle 15°; three averages; scanning time 3:57 min. In vivo, maxilla: isotropic resolution 350 μm; FOV 34 cm³; TR/TE 12/4.8 ms; flip angle 15°; five averages; scanning time 6:40 min. Ex vivo, mandible: isotropic resolution 200 mm³; FOV 39 × 39 × 24 mm (36.5 cm³); TE/TR 4.3/12 ms; 2 averages; flip angle 15°; scanning time 4:38 min.)	Individually fabricated (1 mm diameter copper wire, an adjustable capacitor and crossed diodes) wireless inductively/intraoral. In vivo imaging: 4-cm surface loop coil (Siemens)/close to the cheek opposite to the intraoral coil.	CBCT	2 (dentist > 5 years dedicated to maxillofacial radiology)	Linear measurement and tissue visibility
Fuglsig, 2022^12^	Cadaver specimens (12)	Posterior maxilla and mandible	Geometric accuracy of cortical bone for dental implant planning	9.4 T (Bruker Biospec, Bruker Biospin)	Zero-Echo-Time (FOV 75 mm^3^ isotropic; matrix 3663 pixels; image resolution 0.205 mm; flip angle 0.74°; TR 1.98 ms; bandwidth 2.78 kHz; projection under sampling of two; 50 averages; scan time 6 h)	76-mm quadrature volume coil	CBCT and histology	2 (dentist)	Cortical bone linear measurement
Goto, 2007^35^	Volunteers (2), phantom (1), and dry cadaver (1)	Anterior and posterior mandible	Imaging and geometric accuracy of cortical bone for maxillofacial surgery planning	1.5 T (Symphony, Siemens)	3D Volumetric Interpolated Breath-hold Examination (VIBE) (TR/TE 9.73/3.96 ms; flip angle 20°; voxel 0.7 mm^3^; field of view 173 × 230 mm; scanning time 6.5 min.)	Head coil/NR	CT and micrometer	2 (trained observers)	Cortical bone visualization and linear and angle measurement
Imamura, 2004^9^	Patients (11)	Posterior mandible	Imaging and geometric accuracy of cortical bone for dental implant planning	1.5 T (Shimadzu Corporation)	T1-weighted (TR/TE 500/15 ms, FOV 150 to 260 mm, matrix 256 × 256, number of excitations twice, slice width 2.5 mm perpendicular to the dental arch and overlap 0.5 mm.)	NR	CT	2 (4 years prosthodontist; 27 years prosthodontist)	Mandibular nerve visualization and cortical bone linear measurement

BV/TV, bone-to-volume ratio; CBCT, cone beam computed tomography; CT, computed tomography; DXA, dual-energy X-ray absorptiometry; FOV, field of view; NR, not reported; ST, slice thickness; T, Tesla; TE, echo time; TE, echo time; TR, repetition time; TR, repetition time; MRI, magnetic resonance imaging

### Synthesis of results

A summary of the comparisons between MRI and the reference tests is presented in Table 2. The geometric accuracy of MRI has varied among studies. The deviation in linear measurements ranged from lower values, such as 0.03 mm [34] and 0.04 mm [10], to higher values, reaching 1.67 mm [34] and 3.11 mm [12]. However, MRI measurements showed strong correlations and/or no significant differences compared to histology [10], digital calipers [34, 35] micrometer [35], CT [9, 11, 34], or CBCT [10–12, 33].

**Table 2:** Summary of correlations and deviations between digital and physical magnetic resonance imaging measurements and reference standard tests reported in the included studies

Digital Measurements		
**Correlation**	**Reference test**	**Correlation coefficient (c)**
Choi, 2022^31^	micro-CT	0.943
	CBCT	− 0.068
Cortes, 2018^32^	micro-CT	0.92
Flügge, 2016^10^	CBCT	0.993 (MRI ex vivo)
	CBCT	0.99 (MRI in vivo)
Imamura, 2004^9^	CT	0.687 to 0.868
**Deviation**	**Reference test**	**Mean ± SD (mm)**
Aguiar, 2009^34^	CT	0.03 to 1
Deng, 2014^11^	CT	− 0.011 ± 0.102
Flügge, 2016^10^	CBCT	0.09 (MRI ex vivo)
		0.49 (MRI in vivo)
Imamura, 2004^9^	CT	0 to 0.4
**Physical measurements**		
**Correlation**	**Reference test**	**Correlation coefficient (c)**
Flügge, 2016^10^	Histology	0.99 (MRI ex vivo)
Goto, 2007^35^	Micrometer	0,85 to 0.99
**Deviation**	**Reference test**	**Mean ± SD (mm)**
Aguiar, 2009^34^	Digital caliper	0.13 to 1.67
Deng, 2014^11^	Digital caliper	0.033 ± 0.113
Flügge, 2016^10^	Histology	− 0.04

CT, Computed tomography; CBCT, Cone beam computed tomography.

Bone structural assessments were performed by two studies that measured bone density/volume. Both studies applied high-strength magnetic fields (14T [31] and 15T [32]) and reported a positive correlation between MRI and µCT measurements. However, one study found a weak correlation between MRI and CBCT values [31].

### Risk of bias

**Fig. 2:**
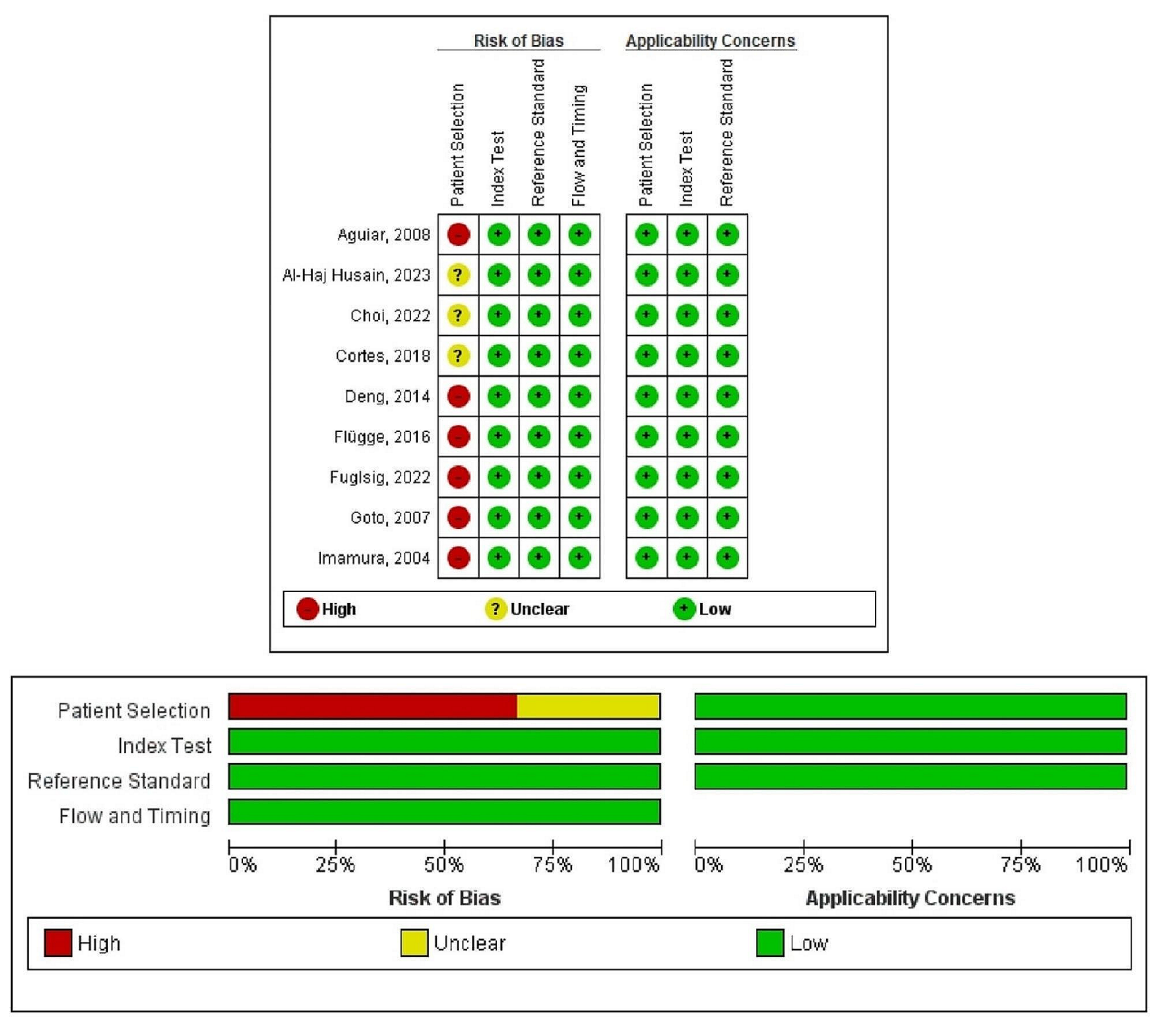
Risk of bias within studies (QUADAS-2).^29^

The QUADAS-2 assessment is shown in Fig. 2. Most studies [9–12, 34, 35] presented a high risk of bias for patient selection due to the lack of clear inclusion and exclusion criteria, whereas three studies [31–33] reported detailed eligibility criteria. The index test, reference standard test, time, and flow were regarded as having a low risk of bias for all included studies. Additionally, all the included studies presented low applicability concerns for patient selection, index tests, and reference standard tests.

### Assessment of the quality of evidence

GRADE assessed certainty as very low due to the high risk of bias for patient selection and heterogeneity across the included studies (Table 3).

**Table 3:** Certainty assessment (GRADE)

Certainty assessment							
No. of studies	Study design	Risk of bias	Indirectness	Inconsistency	Imprecision	Other considerations	Certainty of evidence
9	Cross-sectional	serious^a^	serious ^b^	not serious	serious^c^	none	⊕OOO very low

^a^most studies presented high risk of bias for patient selection.

^b^studies were heterogeneous regarding study design.

^c^studies were heterogeneous regarding index and reference imaging protocols and outcomes evaluated.

## Discussion

This systematic review evaluated whether MRI can be considered a viable method for assessing the jawbone in the maxillofacial region and demonstrated similar results to those of standard reference tests. These findings support the potential of MRI as a valuable tool in the evaluation and diagnosis of jawbone tissues [36] However, the limited number of studies and methodological heterogeneity require careful interpretation, and further studies are needed to establish the comprehensive scope and reliability of MRI in the assessment of maxillofacial bones.

MRI requires a wide range of parameters to assess bone tissues [27], resulting in variability in image quality [37, 38]. These parameters include magnetic field strength and MRI sequences. Therefore, the variations in diagnostic questions among the included studies resulted in variations in the MRI protocols and analysis methods, tailored to the specific objectives of each investigation [39]. One challenging factor in MRI is the rapid signal decay of bone tissue. This factor can be overcome by faster MRI sequences with high signal-to-noise ratios and contrast-to-noise ratios to improve the visualization and segmentation of bone tissue [27] (e.g., 3D VIBE [35], gradient-echo fast low flip angle shots (FLASH) [10], and zero echo time (ZTE)) [12].

In general, MRI reveals image quality, the ability to visualize bone structures [10, 33–35] and consistent quality in defining bone boundaries, as confirmed by high intraobserver and interobserver agreement [34]. For the jawbone, several different MRI sequences were tested to determine the optimal parameters for imaging, focusing on both the examination time and image quality. As a result, the 3D volumetric interpolated breath-hold examination (VIBE) sequence presented the clearest visibility of cortical bone structures [35]. Additionally, other methods for improving jawbone visibility, including the use of dedicated inductively coupled radiofrequency coils attached to oral tissues, which are capable of identifying several maxillofacial structures, including the individual branches of the inferior alveolar nerve, were tested [10].

While the majority of the included studies evaluated dental implant planning, maxillofacial surgery, and diagnosis, a wide range of MRI findings for evaluating the maxillofacial region has also been recently reported [36, 40]. Most included in vitro designs, which exclude clinical factors and lack the complexity of living biological systems. Consequently, the lack of these clinical factors may limit the external validity and generalizability of the findings to real clinical situations, where patient-specific variations are integral to diagnostic and therapeutic decision-making processes.

Variations in study design can introduce inconsistencies in bone measurements and affect the comparability of results across studies. Therefore, the high heterogeneity in imaging protocols and study methodologies restricted quantitative analysis of the findings and resulted in a high risk of bias and very low certainty of evidence. This heterogeneity also limited comparisons between studies and analyses of the reliability, accuracy, and efficacy of MRI for bone assessment in the maxillofacial region. For instance, MRI was compared to different index tests and varied on the outcomes evaluated across studies, potentially leading to different conclusions, or over- and underestimation results.

Like other medical imaging methods, MRI is susceptible to artifacts induced by the presence of various materials within patients [41]. This susceptibility can lead to inadequate visualization or inaccurate assessment of bone structures within regions affected by these artifacts, potentially reducing the accuracy of MRI. However, the impact of these artifacts on accuracy was not explored in most of the included studies. In addition, the exclusion of patients with metallic materials from studies further adds to this limitation, thereby restricting the scope of clinical investigations and their applicability. To address this limitation, improved imaging techniques and postprocessing techniques can be used to mitigate the impact of metallic artifacts and increase the diagnostic value of MRI in this context.

MRI-based assessments of bone morphology are limited by partial volume effects caused by the disparity between MRI resolution and trabecular size, which typically measures 0.1 mm [25]. In cases where the voxel size exceeds this size, trabecular broadening may occur, potentially resulting in the loss or merging of fine trabeculae and thus leading to over- or underestimation [25]. Comparisons with CBCT can also be challenging because of the difference in spatial resolution and image contrast, which consequently impacts the ability to detect fine details of bone morphology and trabecular structure.

During MRI scan planning, balancing the field of view, resolution, signal-to-noise ratio, and acquisition time is essential [42]. However, sequences optimized for bone visualization present unique constraints influenced by factors such as the imaged region’s size and tissue-specific properties like magnetic susceptibility, potentially limiting their utility in certain contexts [42]. Another limitation of the included studies was the scan time, which varied from a few minutes to several hours. Therefore, clinical applications should consider the possibility of motion artifacts. Despite the recent improvements, accessibility, cost, and patient discomfort also should be considered for further clinical application.

Future perspectives on jawbone assessment with MRI include optimizing magnetic fields to improve image quality and resolution; optimizing gradient strength and linearity for faster and more accurate imaging; developing dedicated radiofrequency coils to improve the signal-to-noise ratio and contrast, reduce unwanted signals, and improve patient comfort; refining pulse sequences, especially those dedicated to tissues with fast decaying signals; and improving scanner hardware and software for providing cost-effective image reconstruction and processing [10, 31, 36, 43]. Additionally, the literature still lacks a sensitivity and specificity evaluation of MRI for jawbone assessment [10].

## Conclusions

Limited studies suggest the feasibility of MRI for assessing the jawbone, as MRI provides comparable results to those of standard reference tests. However, further advancements and optimizations are required to increase the applicability, validate the efficacy, and establish the utility of these methods in clinical settings.

### Supplementary Information

The online version contains supplementary material available at.

**Additional file 1:** Search strategies according to the database queried.

**Additional file 2:** Articles excluded and the reasons for exclusion.

## Data Availability

Data described in the manuscript, code book, and analytic code will be made available upon request pending application and approval from the corresponding author.
