# Supplementary material for: Magnetic resonance imaging for jawbone assessment: a systematic review
Source: Head Face Med. 2024 Apr 19;20:25. doi: 10.1186/s13005-024-00424-2 (PMC11027384; doi:10.1186/s13005-024-00424-2)
Supplement: Supplementary file 1 — Additional file 1: Search strategies according to the database queried. [file 13005_2024_424_MOESM1_ESM.docx]

**Additional file 2 -** Articles excluded and the reasons for exclusion.

| **Author, year** | **Reason for exclusion** |  | **Author, year** | **Reason for exclusion** |
| --- | --- | --- | --- | --- |
| 1. Aribandi, 2007 | A |  | 1. Kieselmann, 2018 | C |
| 1. Bohner, 2020 | A |  | 1. Korn, 2015 | A |
| 1. Burian, 2020 | D |  | 1. Korn, 2015b | E |
| 1. Celenk, 2007 | C |  | 1. Korn, 2017 | E |
| 1. Celenk, 2010 | C |  | 1. Krishnasarma, 2017 | E |
| 1. Cevidanes, 2005 | C |  | 1. Kristensen, 2011 | B |
| 1. Chang, 2022 | D |  | 1. Munhoz, 2022 | C |
| 1. Choël, 2004 | D |  | 1. Muraoka, 2022 | C |
| 1. Deepho, 2017 | D |  | 1. Muraoka, 2022b | C |
| 1. Deepho, 2018 | D |  | 1. Ngoc, 2022 | D |
| 1. Ding, 2023 | D |  | 1. Okada, 2022 | C |
| 1. Eggers, 2005 | D |  | 1. Polshina, 2021 | D |
| 1. Elschner, 2017 | A |  | 1. Probst, 2021 | D |
| 1. Elschner, 2017b | E |  | 1. Röttiger, 2019 | D |
| 1. Gaudino, 2010 | E |  | 1. Schoppe, 2017 | D |
| 1. Geibel, 2015 | A |  | 1. Schwindling, 2021 | A |
| 1. Hassfeld, 2013 | D |  | 1. Shishvan, 2018 | D |
| 1. Heil, 2017 | D |  | 1. Speight, 2014 | E |
| 1. Hirahara, 2022b | C |  | 1. Steybe, 2021 | D |
| 1. Hu, 2018 | A |  | 1. Tran, 2023 | D |
| 1. Huber, 2020 | A |  | 1. Tesfai, 2022 | D |
| 1. Isman, 2021 | D |  | 1. Valizadeh, 2016 | D |
| 1. Ito, 2021 | A |  | 1. Voss, 2021 | D |
| 1. Javadrashid, 2015 | D |  | 1. Wang, 2007 | C |
| 1. Javadrashid, 2017 | D |  | 1. Wiesinger, 2018 | D |
| 1. Juerchott, 2018 | D |  | 1. Young, 2018 | B |
| 1. Junhasavasdikul, 2018 | C |  | 1. Yu, 2014 | D |
| 1. Maxilla and mandible sites under deformities, traumatic, pathological, and healing conditions; 2. Lack of magnetic resonance imaging; 3. Lack of reference-standard (histology, physical measurements, or computed tomography); 4. Studies that did not perform quantitative and/or qualitative bone histomorphometry measurements by means of MRI and reference-standard; 5. Reviews, letters, abstract, posters, protocols, personal opinions, case reports, technique articles. | | | | |

**Additional file 2 - References**

1. Aribandi M, Bazan III C. CT and MRI features in Bipolaris fungal sinusitis. Australasian radiology. 2007; 51:127-32.
2. Bohner L, Tortamano P, Meier N, Gremse F, Kleinheinz J, Hanisch M. Trabecular Bone Assessment Using Magnetic-Resonance Imaging: A Pilot Study. International Journal of Environmental Research and Public Health. 2020; 17:9282.
3. Burian E, Sollmann N, Ritschl LM, Palla B, Maier L, Zimmer C, Probst F, Fichter A, Miloro M, Probst M. High resolution MRI for quantitative assessment of inferior alveolar nerve impairment in course of mandible fractures: An imaging feasibility study. Scientific reports. 2020; 10:11566.
4. Celenk C, Celenk P. Evaluation by quantitative magnetic resonance imaging of trabecular bone quality in the dentate and edentulous mandible. Clinical Oral Implants Research. 2008; 19:15-8.
5. Celenk P, Celenk C. Evaluation by quantitative magnetic resonance imaging of trabecular bone quality in mandible and cervical vertebrae. Clinical oral implants research. 2010; 21:409-13.
6. Cevidanes LH, Franco AA, Gerig G, Proffit WR, Slice DE, Enlow DH, Yamashita HK, Kim YJ, Scanavini MA, Vigorito JW. Assessment of mandibular growth and response to orthopedic treatment with 3-dimensional magnetic resonance images. American journal of orthodontics and dentofacial orthopedics. 2005; 128:16-26.
7. Chang MS, Choi JH, Yang IH, An JS, Heo MS, Ahn SJ. Association between condylar bone density and disk displacement in the temporomandibular joint. Journal of Clinical Densitometry. 2022; 25:215-22.
8. Choël L, Last D, Duboeuf F, Seurin MJ, Lissac M, Briguet A, Guillot G. Trabecular alveolar bone microarchitecture in the human mandible using high resolution magnetic resonance imaging. Dentomaxillofacial Radiology. 2004; 33:177-82.
9. Deepho C, Watanabe H, Kotaki S, Sakamoto J, Sumi Y, Kurabayashi T. Utility of fusion volumetric images from computed tomography and magnetic resonance imaging for localizing the mandibular canal. Dentomaxillofacial Radiology. 2017; 46:20160383.
10. Deepho C, Watanabe H, Sakamoto J, Kurabayashi T. Mandibular canal visibility using a plain volumetric interpolated breath-hold examination sequence in MRI. Dentomaxillofacial Radiology. 2018; 47:20170245.
11. Ding WH, Li YF, Liu W, Li W, Wu N, Hu SY, Shi JJ. Effect of occlusal stabilisation splint with or without arthroscopic disc repositioning on condylar bone remodelling in adolescent patients. International Journal of Oral and Maxillofacial Surgery (in press).
12. Eggers G, Rieker M, Fiebach J, Kress B, Dickhaus H, Hassfeld S. Geometric accuracy of magnetic resonance imaging of the mandibular nerve. Dentomaxillofacial Radiology. 2005; 34:285-91.
13. Elschner C, Korn P, Hauptstock M, Schulz MC, Range U, Jünger D, Scheler U. Assessin0067 agreement between preclinical magnetic resonance imaging and histology: An evaluation of their image qualities and quantitative results. PLoS One. 2017; 12:e0179249.
14. Elschner C, Korn P, Hauptstock M, Schulz MC, Range U, Scheler U. Magnetic resonance imaging versus histology – Do they really measure the same? In: Session 31: Imaging and image processing III–Nanoparticle imaging and MRI. Biomedical Engineering/Biomedizinische Technik. 2017; 62:s271-4.
15. Gaudino, C. Csernus, R, Heiland, S. Pham, M. Cosgarea, R. Bendszus, M. Rohde, S. Comparative evaluation of Magnetic Resonance Imaging and Multislice Computed Tomography in the imaging of dental and periodontal structures: a subjective image quality in-vitro study. In, Clin Neuroradiol. 2010; 20:189–220
16. Geibel MA, Schreiber ES, Bracher AK, Hell E, Ulrici J, Sailer LK, Ozpeynirci Y, Rasche V. Assessment of apical periodontitis by MRI: a feasibility study. InRöFo-Fortschritte auf dem Gebiet der Röntgenstrahlen und der bildgebenden Verfahren 2015 Apr (Vol. 187, No. 04, pp. 269-275). © Georg Thieme Verlag KG.
17. Hassfeld S, Fiebach J, Widmann S, Heiland S, Mühling J. Magnetic resonance tomography for planning dental implantation. Mund-, Kiefer-und Gesichtschirurgie: MKG. 2001; 5:186-92.
18. Heil A, Lazo Gonzalez E, Hilgenfeld T, Kickingereder P, Bendszus M, Heiland S, Ozga AK, Sommer A, Lux CJ, Zingler S. Lateral cephalometric analysis for treatment planning in orthodontics based on MRI compared with radiographs: A feasibility study in children and adolescents. PloS one. 2017; 12:e0174524.
19. Hirahara N, Kaneda T, Muraoka H, Ito K, Okada S, Tokunaga S. Quantitative assessment of the mandibular condyle in patients with rheumatoid arthritis using diffusion-weighted imaging. Journal of Oral and Maxillofacial Surgery. 2021; 79:546-50.
20. Hu S, Zhou Y, Zhao Y, Xu Y, Zhang F, Gu N, Ma J, Reynolds MA, Xia Y, Xu HH. Enhanced bone regeneration and visual monitoring via superparamagnetic iron oxide nanoparticle scaffold in rats. Journal of Tissue Engineering and Regenerative Medicine. 2018; 12:e2085-98.
21. Huber FA, Schumann P, Von Spiczak J, Wurnig MC, Klarhöfer M, Finkenstaedt T, Bedogni A, Guggenberger R. Medication-Related osteonecrosis of the Jaw—Comparison of bone imaging using ultrashort Echo-Time magnetic resonance imaging and cone-beam computed tomography. Investigative radiology. 2020 ; 55:160-7.
22. Isman O, Isman E. Identification of various orthodontic materials as foreign bodies via panoramic radiography, cone beam computed tomography, magnetic resonance imaging, and ultrasonography: an in vitro study. Oral Radiology. 2021; 37:524-30.
23. Ito K, Muraoka H, Hirahara N, Sawada E, Hirohata S, Otsuka K, Okada S, Kaneda T. Quantitative assessment of mandibular bone marrow using computed tomography texture analysis for detect stage 0 medication-related osteonecrosis of the jaw. European Journal of Radiology. 2021; 145:110030.
24. Javadrashid R, Fouladi DF, Golamian M, Hajalioghli P, Daghighi MH, Shahmorady Z, Niknejad MT. Visibility of different foreign bodies in the maxillofacial region using plain radiography, CT, MRI and ultrasonography: an in vitro study. Dentomaxillofacial Radiology. 2015; 44:20140229.
25. Javadrashid R, Golamian M, Shahrzad M, Hajalioghli P, Shahmorady Z, Fouladi DF, Sadrarhami S, Akhoundzadeh L. Visibility of different intraorbital foreign bodies using plain radiography, computed tomography, magnetic resonance imaging, and cone-beam computed tomography: an in vitro study. Canadian Association of Radiologists Journal. 2017; 68:194-201.
26. Juerchott A, Saleem MA, Hilgenfeld T, Freudlsperger C, Zingler S, Lux CJ, Bendszus M, Heiland S. 3D cephalometric analysis using magnetic resonance imaging: validation of accuracy and reproducibility. Scientific Reports. 2018; 8:1-1.
27. Junhasavasdikul T, Abadeh A, Tolend M, Doria AS. Developing a reference MRI database for temporomandibular joints in healthy children and adolescents. Pediatric Radiology. 2018; 48:1113-22.
28. Kieselmann JP, Kamerling CP, Burgos N, Menten MJ, Fuller CD, Nill S, Cardoso MJ, Oelfke U. Geometric and dosimetric evaluations of atlas-based segmentation methods of MR images in the head and neck region. Physics in Medicine & Biology. 2018; 63:145007.
29. Korn P, Elschner C, Hautstock M, Range U, Pradel W, Lauer G. Method comparison between bone histology and magnetic resonance imaging: is magnetic resonance imaging suitable to quantify osteogenesis?. International Journal of Oral and Maxillofacial Surgery. 2017; 46:208.
30. Korn P, Elschner C, Schulz MC, Range U, Mai R, Scheler U. MRI and dental implantology: two which do not exclude each other. Biomaterials. 2015; 53:634-45.
31. Korn P, Elschner C, Schulz MC, Range U, Mai R, Scheler U. Titanium coated peek implants as basis for multimodal imaging in implant research. International Journal of Oral and Maxillofacial Surgery. 2015; 44:e94.
32. Krishnasarma, R. Predicting difficult airway at birth in micrognathia: A retrospective review of fetal MRI and postnatal outcomes. In, SPR 2017. Pediatr Radiol 47 (Suppl 1), 1–296 (2017)
33. Kristensen KD, Hauge EM, Dalstra M, Stoustrup P, Küseler A, Pedersen TK, Herlin T. Association between condylar morphology and changes in bony microstructure and sub‐synovial inflammation in experimental temporomandibular joint arthritis. Journal of oral pathology & medicine. 2011; 40:111-20.
34. Munhoz L, Abdala Júnior R, Choi IG, Arita ES. Diffusion-weighted magnetic resonance imaging of mandibular bone marrow: do apparent diffusion coefficient values of the cervical vertebrae and mandible correlate with age?. Oral Radiology. 2022: 1-8.
35. Muraoka H, Ito K, Hirahara N, Ichiki S, Kondo T, Kaneda T. Magnetic resonance imaging texture analysis in the quantitative evaluation of acute osteomyelitis of the mandibular bone. Dentomaxillofacial Radiology. 2022; 51:20210321.
36. Muraoka H, Ito K, Hirahara N, Okada S, Kondo T, Kaneda T. Quantitative assessment of age-related changes in the mandibular bone marrow using apparent coefficient value. Oral Radiology. 2022: 1-6.
37. Ngoc TT, Wang DH, Yang MC, Chen JC, Wu PH, Yang CC, Hsu WE, Hsu ML. Effects of food hardness on temporomandibular joint osteoarthritis: Qualitative and quantitative micro-CT analysis of rats in vivo. In, Annals of Anatomy-Anatomischer Anzeiger. 2022; 23:152029.
38. Okada S, Ito K, Muraoka H, Hirahara N, Itakura G, Ichiki S, Komatsu T, Kondo T, Kaneda T. Quantitative assessment of the mandibular bone marrow of diabetes mellitus patients using diffusion-weighted magnetic resonance imaging. Oral Radiology. 2022; 1:1-7.
39. Polshina VI, Reshetov IV, Serova NS, Babkova AA, Lisavin AA, Semenov PJ, Roschina AV. Comprehensive radiological diagnossis in patients with temporomandibular joint dysfunction (TMD). Russian electronic journal of radiological diagnostics. 2021; 11:88-102.
40. Probst FA, Burian E, Malenova Y, Lyutskanova P, Stumbaum MJ, Ritschl LM, Kronthaler S, Karampinos D, Probst M. Geometric accuracy of magnetic resonance imaging–derived virtual 3‐dimensional bone surface models of the mandible in comparison to computed tomography and cone beam computed tomography: A porcine cadaver study. Clinical Implant Dentistry and Related Research. 2021; 23:779-88.
41. Röttiger C, Hellige M, Ohnesorge B, Bienert-Zeit A. Magnetic resonance imaging and computed tomography of equine cheek teeth and adjacent structures: comparative study of image quality in horses in vivo, post-mortem and frozen-thawed. Acta Veterinaria Scandinavica. 2019; 61:1-4.
42. Schoppe C, Hellige M, Rohn K, Ohnesorge B, Bienert-Zeit A. Comparison of computed tomography and high-field (3.0 T) magnetic resonance imaging of age-related variances in selected equine maxillary cheek teeth and adjacent tissues. BMC veterinary research. 2017; 13:1-2.
43. Schwindling FS, Juerchott A, Boehm S, Rues S, Kronsteiner D, Heiland S, Bendszus M, Rammelsberg P, Hilgenfeld T. Three‐dimensional accuracy of partially guided implant surgery based on dental magnetic resonance imaging. Clinical Oral Implants Research. 2021; 32:1218-27.
44. Shishvan HH, Ebrahimnejad H. A study on the ability of panoramic, CT, Cone-beam CT, MRI and ultrasonography in detecting different foreign-bodies in the maxillofacial region (an in-vitro study). Electron J Gen Med. 2018; 15:em16.
45. Speight R, Perkinson A, Smith D, Sykes J, Prestwich R, Sen M, Ramasamy S, Wright S, Selvan A. PO-0907: Comparison of quantitative and clinical assessment of deformable image registration of CT and MRI for H&N patients. In, Radiotherapy and Oncology. 2014(111):S109.
46. Steybe D, Russe MF, Ludwig U, Sprave T, Vach K, Semper-Hogg W, Schmelzeisen R, Voss PJ, Poxleitner P. Intraoperative marking of the tumour resection surface for improved radiation therapy planning in head and neck cancer: preclinical evaluation of a novel liquid fiducial marker. Dentomaxillofacial Radiology. 2021; 50:20200290.
47. Tran TT, Wang DH, Yang MC, Chen JC, Wu PH, Yang CC, Hsu WE, Hsu ML. Effects of food hardness on temporomandibular joint osteoarthritis: Qualitative and quantitative micro-CT analysis of rats in vivo. Annals of Anatomy-Anatomischer Anzeiger. 2023; 246:152029.
48. Tesfai AS, Vollmer A, Özen AC, Braig M, Semper-Hogg W, Altenburger MJ, Ludwig U, Bock M. Inductively coupled intraoral flexible coil for increased visibility of dental root canals in magnetic resonance imaging. Investigative radiology. 2022; 57(3):163-70.
49. Valizadeh S, Pouraliakbar H, Kiani L, Safi Y, Alibakhshi L. Evaluation of visibility of foreign bodies in the maxillofacial region: comparison of computed tomography, cone beam computed tomography, ultrasound and magnetic resonance imaging. Iranian Journal of Radiology. 2016; 13(4).
50. Voss JO, Doll C, Raguse JD, Beck-Broichsitter B, Walter-Rittel T, Kahn J, Böning G, Maier C, Thieme N. Detectability of foreign body materials using X-ray, computed tomography and magnetic resonance imaging: A phantom study. European Journal of Radiology. 2021; 135:109505.
51. Wang HY, Shih TT, Wang JS, Shiau YY, Chen YJ. Low bone mineral density and temporomandibular joint derangement in young females. Journal of Orofacial Pain. 2007; 21(2).
52. Wiesinger F, Bylund M, Yang J, Kaushik S, Shanbhag D, Ahn S, Jonsson JH, Lundman JA, Hope T, Nyholm T, Larson P. Zero TE‐based pseudo‐CT image conversion in the head and its application in PET/MR attenuation correction and MR‐guided radiation therapy planning. Magnetic resonance in medicine. 2018; 80:1440-51.
53. Yang BT, Wang ZC, Liu S, Xian JF, Liu ZL, Lan BS. CT and MRI diagnosis of cavernous hemangioma in paranasal sinus. Chinese Journal of Radiology. 2000.
54. Yu H, Caldwell C, Balogh J, Mah K. Toward magnetic resonance–only simulation: segmentation of bone in MR for radiation therapy verification of the head. International Journal of Radiation Oncology* Biology* Physics. 2014; 89:649-5.
