# Supplementary material for: Magnetic resonance imaging for jawbone assessment: a systematic review
Source: Head Face Med. 2024 Apr 19;20:25. doi: 10.1186/s13005-024-00424-2 (PMC11027384; doi:10.1186/s13005-024-00424-2)
Supplement: Supplementary file 2 — Additional file 2: Articles excluded and the reasons for exclusion. [file 13005_2024_424_MOESM2_ESM.docx]

**Additional file 1**. Search strategies according to the database queried.

| Database | Query | Items found |
| --- | --- | --- |
| Pubmed | ("maxillofacial" OR jaw [MeSH] OR "jaw" OR dentistry [MeSH] OR “dentistry” OR "oral surgery" OR maxilla [MeSH] OR "maxilla" OR "maxillary" OR mandible [MeSH] OR "mandible" OR surgery oral [MeSH] OR "surgery oral" OR mouth [MeSH] OR "mouth" OR "head and neck" OR "preoperative planning" OR Temporomandibular Joint [MeSH] OR "temporomandibular joint" OR “TMJ” OR Alveolar Process [MeSH] OR "alveolar process" OR "alveolar bone" OR "alveolar ridge" OR "intraoral" OR "jawbones") AND (magnetic resonance imaging [MeSH] OR "magnetic resonance imaging" OR "nuclear magnetic resonance imaging" OR "magnetic resonance" OR "MRI" OR "NMRI" OR “PET-MRI”) AND ("bone morphometric" OR "bone morphometry" OR "bone analys*" OR "bone assessment" OR "bone morphology" OR "trabecular bone volume" OR "cortical bone volume" OR "bone volume" OR "trabecular volume fraction" OR "bone specific surface" OR "trabecular thickness" OR "trabecular separation" OR "bone porosity" OR "bone quality" OR "bone measurement" OR "bone quality assessment" OR "bone quantitative" OR Bone Density [MeSH] OR "bone density" OR "bone mineral density" OR "bone densitometry" OR "qualitative assessment" OR "quantitative assessment" OR "visibility" OR "quantitative image analysis" OR "geometric accuracy" OR "dimensional accuracy") | 333 |
| EMBASE | ('maxillofacial' OR 'jaw' OR 'dentistry' OR 'oral surgery' OR 'maxilla' OR 'maxillary' OR 'mandible' OR 'oral surgery' OR 'mouth' OR 'head and neck' OR 'preoperative planning' OR 'temporomandibular joint' OR 'TMJ' OR 'alveolar process' OR 'alveolar bone' OR 'alveolar ridge' OR 'intraoral' OR 'jawbones') AND ('magnetic resonance imaging' OR 'nuclear magnetic resonance imaging' OR 'magnetic resonance' OR 'MRI' OR 'NMRI' OR 'PET-MRI') AND ('bone morphometric' OR 'bone morphometry' OR 'bone analys*' OR 'bone assessment' OR 'bone morphology' OR 'trabecular bone volume' OR 'cortical bone volume' OR 'bone volume' OR 'trabecular volume fraction' OR 'bone specific surface' OR 'trabecular thickness' OR 'trabecular separation' OR 'bone porosity' OR 'bone quality' OR 'bone measurement' OR 'bone quality assessment' OR 'bone quantitative' OR 'bone density' OR 'bone mineral density' OR 'bone densitometry' OR 'qualitative assessment' OR 'quantitative assessment' OR visibility OR 'quantitative image analysis' OR 'geometric accuracy' OR 'dimensional accuracy') | 657 |
| Scopus | TITLE-ABS-KEY("maxillofacial" OR "jaw" OR "dentistry" OR "oral surgery" OR "maxilla" OR "maxillary" OR "mandible" OR "mouth" OR "head and neck" OR "preoperative planning" OR "temporomandibular joint" OR “TMJ” OR "alveolar process" OR "alveolar bone" OR "alveolar ridge" OR "intraoral" OR "jawbones") AND TITLE-ABS-KEY("magnetic resonance imaging" OR "nuclear magnetic resonance imaging" OR "magnetic resonance" OR "MRI" OR "NMRI" OR “PET-MRI”) AND TITLE-ABS-KEY("bone morphometric" OR "bone morphometry" OR "bone analys*" OR "bone assessment" OR "bone morphology" OR "trabecular bone volume" OR "cortical bone volume" OR "bone volume" OR "trabecular volume fraction" OR "bone specific surface" OR "trabecular thickness" OR "trabecular separation" OR "bone porosity" OR "bone quality" OR "bone measurement" OR "bone quality assessment" OR "bone quantitative" OR "bone density" OR "bone mineral density" OR "bone densitometry" OR "qualitative assessment" OR "quantitative assessment" OR "visibility" OR "quantitative image analysis" OR "geometric accuracy" OR "dimensional accuracy") | 457 |
| Web of Science | ((ALL=("maxillofacial" OR "jaw" OR "dentistry" OR "oral surgery" OR "maxilla" OR "maxillary" OR "mandible" OR "mouth" OR "head and neck" OR "preoperative planning" OR "temporomandibular joint" OR “TMJ” OR "alveolar process" OR "alveolar bone" OR "alveolar ridge" OR "intraoral" OR "jawbone")) AND ALL="magnetic resonance imaging" OR "nuclear magnetic resonance imaging" OR "magnetic resonance" OR "MRI" OR "NMRI" OR “PET-MRI”)) AND ALL=("bone morphometric" OR "bone morphometry" OR "bone analys*" OR "bone assessment" OR "bone morphology" OR "trabecular bone volume" OR "cortical bone volume" OR "bone volume" OR "trabecular volume fraction" OR "bone specific surface" OR "trabecular thickness" OR "trabecular separation" OR "bone porosity" OR "bone quality" OR "bone measurement" OR "bone quality assessment" OR "bone quantitative" OR "bone density" OR "bone mineral density" OR "bone densitometry" OR "qualitative assessment" OR "quantitative assessment" OR "visibility" OR "quantitative image analysis" OR "geometric accuracy" OR "dimensional accuracy") | 211 |
| Cochrane | ("maxillofacial" OR "jaw" OR "dentistry" OR "oral surgery" OR "maxilla" OR "maxillary" OR "mandible" OR "mouth" OR "head and neck" OR "preoperative planning" OR "temporomandibular joint" OR “TMJ” OR "alveolar process" OR "alveolar bone" OR "alveolar ridge" OR "intraoral" OR "jawbones") in Title Abstract Keyword AND ("magnetic resonance imaging" OR "nuclear magnetic resonance imaging" OR "magnetic resonance" OR "MRI" OR "NMRI" OR “PET-MRI”) in Title Abstract Keyword AND ("bone morphometric" OR "bone morphometry" OR "bone analys" OR "bone assessment" OR "bone morphology" OR "trabecular bone volume" OR "cortical bone volume" OR "bone volume" OR "trabecular volume fraction" OR "bone specific surface" OR "trabecular thickness" OR "trabecular separation" OR "bone porosity" OR "bone quality" OR "bone measurement" OR "bone quality assessment" OR "bone quantitative" OR "bone density" OR "bone mineral density" OR "bone densitometry" OR "qualitative assessment" OR "quantitative assessment" OR "visibility" OR "quantitative image analysis" OR "geometric accuracy" OR "dimensional accuracy”) in Title Abstract Keyword - (Word variations have been searched) | 25 |
